# MELHAC Improves Glucose and Lipid Metabolism in HFD + Alloxan-Induced Mice

**DOI:** 10.3390/nu18132145

**Published:** 2026-07-02

**Authors:** Zihao Wang, Yang Yang, Zhixi Geng, Senyang Hu, Wenhua Jin, Hejing Tang, Jianmin Zou, Chang Liu, Yinhua Zhu

**Affiliations:** 1 Beijing Advanced Innovation Center for Food Nutrition and Human Health, Department of Nutrition and Health, China Agricultural University, Beijing 100193, China; wangzihao2025@cau.edu.cn (Z.W.); yyang@cau.edu.cn (Y.Y.);; 2 Institute of Environment and Sustainable Development in Agriculture, Chinese Academy of Agricuitural Sciences, Key Laboratory of Agricultural Environment, MARA, Beijing 100081, China

**Keywords:** glucose and lipid metabolism, hepatic oxidative stress, gut microbiota, high-fat diet plus alloxan, plant extract mixture, metabolomics, safety evaluation

## Abstract

**Background:** Glucose and lipid metabolism disorders are characterized by hyperglycemia, dyslipidemia, hepatic oxidative stress, lipid accumulation, and gut microbiota dysbiosis, all of which contribute to progressive metabolic dysfunction and tissue injury. As a plant extract mixture derived from mulberry leaves, lotus leaves, and Eucommia leaves, MELHAC (Mulberry–Eucommia–Lotus Herbal Aqueous Complex) was developed as a medicinal and edible formula with potential multi-component metabolic regulatory activity. In the present study, we systematically evaluated the effects of MELHAC on glucose and lipid metabolic abnormalities in high-fat diet (HFD) plus alloxan-induced mice. **Methods:** The phytochemical profile of MELHAC was characterized using untargeted LC–MS and network pharmacology. Its metabolic effects were evaluated in HFD plus alloxan-induced mice by measuring fasting blood glucose, serum lipid parameters, glucose tolerance, hepatic oxidative stress markers, histopathological changes, hepatic lipid accumulation, gut microbiota composition, and preliminary safety indices. **Results**: Chemical characterization revealed that MELHAC contains abundant bioactive constituents dominated by flavonoids, phenolic acids and alkaloids. In vivo experiments demonstrated that MELHAC lowered fasting blood glucose, total cholesterol and triglyceride levels, while ameliorating glucose intolerance and pathological damage in the liver, kidney and pancreas. MELHAC also improved liver-related biochemical abnormalities, increased hepatic superoxide dismutase, decreased malondialdehyde, and reduced hepatic lipid accumulation, indicating protective effects against oxidative stress and steatosis associated with metabolic dysfunction. In addition, MELHAC modulated gut microbial community structure and differential taxa linked to metabolic homeostasis. Short-term high-dose administration did not cause obvious abnormalities in serum biochemical, hematological, or histopathological indices. **Conclusions:** These findings suggest that MELHAC has potential as a plant-derived functional ingredient for improving glucose and lipid metabolic disorders and may provide an experimental basis for the future development of functional foods targeting metabolic health.

## 1. Introduction

Disorders of glucose and lipid metabolism, including obesity, type 2 diabetes mellitus (T2DM), dyslipidemia, and non-alcoholic fatty liver disease (NAFLD), have become major global health challenges [1,2]. These metabolic abnormalities are closely associated with increased risks of cardiovascular disease, chronic kidney disease, hepatic injury, and other long-term complications, thereby imposing substantial burdens on public health systems and quality of life [3,4,5]. At the pathological level, glucose and lipid metabolism disorders are typically characterized by hyperglycemia, abnormal lipid profiles, insulin resistance, ectopic lipid deposition, and chronic low-grade inflammation [5,6,7,8,9,10]. Among the affected organs, the liver plays a central role in maintaining systemic metabolic homeostasis, and hepatic steatosis is not only a hallmark of metabolic dysfunction but also an important driver of disease progression [3,4,5,6,7,8].

The mechanisms underlying glucose and lipid metabolism disorders are complex and multifactorial. Excessive nutrient intake, especially chronic high-fat feeding, can disturb hepatic lipid handling and glucose utilization, leading to increased de novo lipogenesis, reduced fatty acid oxidation, oxidative stress, mitochondrial dysfunction, and impaired insulin signaling [8,9,10,11,12,13,14,15]. These alterations can further promote hepatic lipid accumulation and aggravate systemic metabolic imbalance [9,10,11,12,13,14,15]. In addition, increasing evidence has shown that gut microbiota dysbiosis is closely linked to host glucose and lipid metabolism through multiple pathways, including short-chain fatty acid production, bile acid metabolism, inflammatory regulation, and intestinal barrier integrity [16,17,18,19,20,21,22,23]. Therefore, effective interventions for glucose and lipid metabolism disorders should ideally target not only circulating metabolic parameters but also hepatic injury, oxidative stress, and gut microbial imbalance.

Current strategies for the prevention and management of glucose and lipid metabolism disorders mainly include lifestyle intervention, dietary regulation, and pharmacological treatment [2,3,4,5,6,7,15]. Although drugs such as metformin, lipid-lowering agents, and insulin sensitizers play important therapeutic roles, their long-term use may be limited by side effects, cost, poor compliance, or the inability to simultaneously regulate multiple pathological processes [2,3]. Lifestyle and dietary interventions are fundamental but often difficult to maintain over time [1,2]. These limitations have stimulated growing interest in food-derived and plant-based interventions that may provide broader metabolic benefits with better tolerability and greater suitability for long-term use [15].

In recent years, medicinal and edible plant materials have attracted considerable attention as potential regulators of metabolic health. Many plant-derived extracts and phytochemicals, including flavonoids, phenolic acids, alkaloids, and polysaccharides, have been reported to exert hypoglycemic, hypolipidemic, antioxidant, anti-inflammatory, and hepatoprotective effects [15,24,25,26,27,28,29,30,31,32,33,34,35,36,37,38]. Mulberry (*Morus alba* L.) leaves are rich in flavonoids, phenolic acids, and alkaloids such as 1-deoxynojirimycin and have been widely studied for their antihyperglycemic and gut microbiota-modulating activities [24,25,26,27]. Lotus (*Nelumbo nucifera* Gaertn.) leaves contain alkaloids, flavonoids, and phenolic compounds and have shown potential in improving obesity, lipid metabolism, and inflammation [28,29,30,31]. Eucommia (*Eucommia ulmoides* Oliver) leaves are also rich in chlorogenic acid, flavonoids, and related metabolites and have been associated with antioxidant, hepatoprotective, hypoglycemic, and hypolipidemic effects [32,33,34,35,36,37,38]. These findings suggest that medicinal and edible plants may provide multiple bioactive components capable of regulating several aspects of metabolic dysfunction.

However, most existing studies have focused on single plant extracts or isolated compounds, whereas the biological effects of mixed plant-derived formulations remain insufficiently understood [15,24,25,26,27,28,29,30,31,32,33,34,35,36,37,38]. In practice, medicinal and edible products are often consumed as decoctions, soups, powders, or composite formulations rather than as purified monomers. Compared with single compounds, such mixed systems may better reflect real dietary exposure and may exert synergistic or complementary effects through multi-component and multi-target interactions [15,24,25,26,27,28,29,30,31,32,33,34,35,36,37,38]. At the same time, the complexity of such mixtures also makes their effective material basis and mechanisms more difficult to define. Therefore, there is still a need for systematic studies combining chemical characterization, mechanism prediction, in vivo efficacy evaluation, and safety assessment to determine whether plant extract mixtures can effectively ameliorate glucose and lipid metabolism disorders.

From the perspective of product development, medicinal and edible formulations also offer several practical advantages. Owing to their food-compatible origin, they may have relatively favorable safety profiles, better acceptability, and greater potential for long-term consumption compared with conventional drugs [24,25,26,27,28,29,30,31,32,33,34,35,36,37,38]. More importantly, such products may be applied not only in metabolic intervention but also in early nutritional prevention and health maintenance. This makes medicinal and edible plant mixtures promising candidates for the development of functional foods targeting metabolic health.

Based on these considerations, we prepared MELHAC, a plant extract mixture derived from mulberry leaves, lotus leaves, and Eucommia leaves, and evaluated its regulatory effects in a high-fat diet plus alloxan-induced mouse model of glucose and lipid metabolism disorder. In the present study, untargeted LC–MS, network pharmacology, animal experiments, gut microbiota analysis, and preliminary safety evaluation were integrated to investigate the chemical basis, metabolic efficacy, and potential mechanisms of MELHAC. This study aimed to provide experimental evidence for the potential application of MELHAC as a medicinal and edible functional ingredient for improving glucose and lipid metabolism disorders.

## 2. Materials and Methods

### 2.1. Preparation of the Herbal Formula

“Mulberry leaves *(Morus alba* L.), lotus leaves (*Nelumbo nucifera* Gaertn.), and Eucommia leaves (*Eucommia ulmoides* Oliver) were obtained as commercial medicinal-grade products from an established domestic supplier via e-commerce platform (Taobao, Alibaba, Hangzhou, China) and were verified to comply with the standard botanical characteristics. After drying and pulverization, the plant materials were extracted twice with distilled water at a solvent-to-plant material ratio of 10:1 (*v*/*w*) for 2 h each cycle under conditions of a vacuum of −0.06 to −0.09 MPa and a temperature of 65 °C”. The powdered extracts of mulberry leaves, lotus leaves, and Eucommia leaves were thoroughly mixed at a specific weight ratio of 1:1:1. To ensure homogeneity, the combined powders were mechanically blended using a geometric dilution method and passed through a 80-mesh sieve three times to obtain a uniform composite powder (MELHAC). The composite mixture was stored in a desiccator at room temperature prior to use. Before administration, the formula was freshly dissolved in distilled water to the required concentrations.

### 2.2. Animals and Experimental Design

A total of 30 adult male C57BL/6 mice (aged between 5 and 7 weeks, weighing 19–30 g) were provided by Beijing Huafukang Biotechnology Co., Ltd. (Beijing, China). The mice were housed under controlled conditions (temperature 22 ± 2 °C, light/dark cycle 12 h), and had free access to standard feed and high-fat feed. Each group (*n* = 5) of mice was acclimated to the environment for one week before the start of the experiment. All operations related to the mice were approved by the Institutional Animal Care and Use Committee of China Agricultural University (protocol number: AW40115202-5-501; 4 November 2025).

The normal control group was fed a standard chow diet throughout the experiment. The other groups were fed a high-fat diet (HFD) to induce metabolic disturbance. After 4 weeks of HFD feeding, mice in the model, positive control, and treatment groups were intraperitoneally injected with alloxan at a dose of 125–130 mg/kg BW.ip to establish the HFD + alloxan-induced model. After model establishment, mice in the positive control group received metformin HCl at 200 mg/kg/d, while mice in the treatment group received the herbal formula at 166.7 mg/kg body weight by oral gavage once daily for 2 weeks. Mice in the normal control and model groups received an equal volume of distilled water.

The sample size was determined based on previous experience with similar animal studies, practical feasibility, and the 3R principle of reducing animal use. Five animals were included in each experimental group. No formal a priori sample size calculation or power analysis was performed.

### 2.3. Experimental Design for Prophylactic Study

At the end of the experiment, mice were fasted overnight and deeply anesthetized with sodium pentobarbital prior to terminal blood collection. Blood samples were collected by cardiac puncture and centrifuged at 12,000× *g* for 10 min at 4 °C to obtain serum. Following blood collection, mice were euthanized by cervical dislocation under deep anesthesia to ensure death. The liver, kidney, pancreas, lung, and heart were then rapidly excised and rinsed with cold saline when necessary. Tissue samples were either fixed in 4% paraformaldehyde logical analysis or snap-frozen in liquid nitrogen and stored at −80 °C for subsequent biochemical and molecular analyses.

### 2.4. Histopathological Examination and Oil Red O Staining

For histopathological examination, liver tissues fixed in 4% paraformaldehyde were dehydrated, embedded in paraffin, sectioned, and stained with hematoxylin and eosin (H&E). Histological changes, including hepatocyte arrangement, vacuolar degeneration, and inflammatory infiltration, were observed under a light microscope.

For lipid deposition analysis, frozen liver sections were prepared and stained with Oil Red O. For Oil Red O quantification, stained sections were imaged under identical microscope settings at 200× magnification. Images were analyzed using ImageJ software (version 1.54p, National Institutes of Health, Bethesda, MD, USA). The positive staining area was quantified using a uniform threshold applied to all images within the same experiment.

### 2.5. Determination of Serum Biochemical Parameters

Serum biochemical parameters related to glucose and lipid metabolism were determined using commercial assay kits according to the manufacturers’ instructions. The measured parameters included fasting blood glucose, total cholesterol (TC), triglycerides (TG), low-density lipoprotein cholesterol (LDL-C), and high-density lipoprotein cholesterol (HDL-C). In addition, serum alanine aminotransferase (ALT) and aspartate aminotransferase (AST) levels were measured to assess liver function.

### 2.6. Determination of Hepatic Oxidative Stress Markers

Liver tissues were homogenized in ice-cold physiological saline to prepare tissue homogenates. After centrifugation, the supernatants were collected for biochemical analysis. The activities of superoxide dismutase (SOD) as well as the level of malondialdehyde (MDA) were determined using commercial assay kits according to the manufacturers’ protocols.

### 2.7. Statistical Analysis

All data were expressed as mean ± standard error of the mean (SEM). Statistical analysis was performed using GraphPad Prism 10.1.2. Differences among multiple groups were analyzed using one-way analysis of variance (ANOVA), followed by Dunnett’s test. A value of *p* < 0.05 was considered statistically significant.

### 2.8. LC-MS Analysis

The phytochemical profile of MELHAC was characterized using ultra-high-performance liquid chromatography coupled with high-resolution mass spectrometry (UHPLC-Q Exactive HF-X, Thermo Fisher Scientific, Waltham, MA, USA).

Briefly, MELHAC powder was dissolved in 80% methanol at a concentration of 1 mg/mL and vortexed thoroughly for 5 min. The solution was sonicated for 30 min at room temperature and centrifuged at 13,000× *g* for 15 min. The resulting supernatant was filtered through a 0.22 μm membrane filter and transferred into LC–MS vials for analysis. Quality control (QC) samples were prepared by pooling equal volumes of all extracts and were injected periodically throughout the analytical sequence to monitor instrument stability.

Chromatographic separation was performed on an ACQUITY UPLC BEH C18 column (100 mm × 2.1 mm, 1.7 μm; Waters, Milford, MA, USA). The mobile phase consisted of solvent A (2% acetonitrile in water containing 0.1% formic acid) and solvent B (acetonitrile containing 0.1% formic acid). The injection volume was 1 μL and the column temperature was maintained at 40 °C. Mass spectrometric data were acquired in both positive and negative electrospray ionization modes (ESI+ and ESI−). The scan range was *m*/*z* 70–1050.

Raw data were processed using Progenesis QI v3.0 (Waters Corporation, Milford, MA, USA). Metabolite annotation was performed by matching MS and MS/MS spectra against the Majorbio Plant Metabolite Database (MJDBPM), with a mass accuracy threshold of less than 10 ppm.

### 2.9. Gut Microbiota Analysis

Intestinal content samples were collected at the end of the experiment and stored at −80 °C until analysis. Gut microbiota sequencing was performed by Shanghai Majorbio Bio-Pharm Technology Co., Ltd. (Shanghai, China). Microbial genomic DNA was extracted and the bacterial 16S rRNA gene was amplified using barcode-tagged primers targeting the designated sequencing region. Sequencing libraries were constructed and sequenced on an Illumina platform.

Raw reads were quality-filtered, merged, and denoised using the DADA2/Deblur pipeline to generate amplicon sequence variants (ASVs). Taxonomic annotation was performed based on representative ASV sequences. Alpha diversity was evaluated using the Shannon index, while beta diversity was assessed by principal component analysis (PCA) and principal coordinate analysis (PCoA). Differences in microbial community composition were analyzed at the phylum and genus levels. Differentially abundant taxa were identified using linear discriminant analysis effect size (LEfSe). Bioinformatic analyses and data visualization were performed using the Majorbio Cloud Platform (https://cloud.majorbio.com/; accessed on 3 April 2026).

## 3. Results

### 3.1. MELHAC Mass Spectrometry Analysis Results

**Figure 1:**
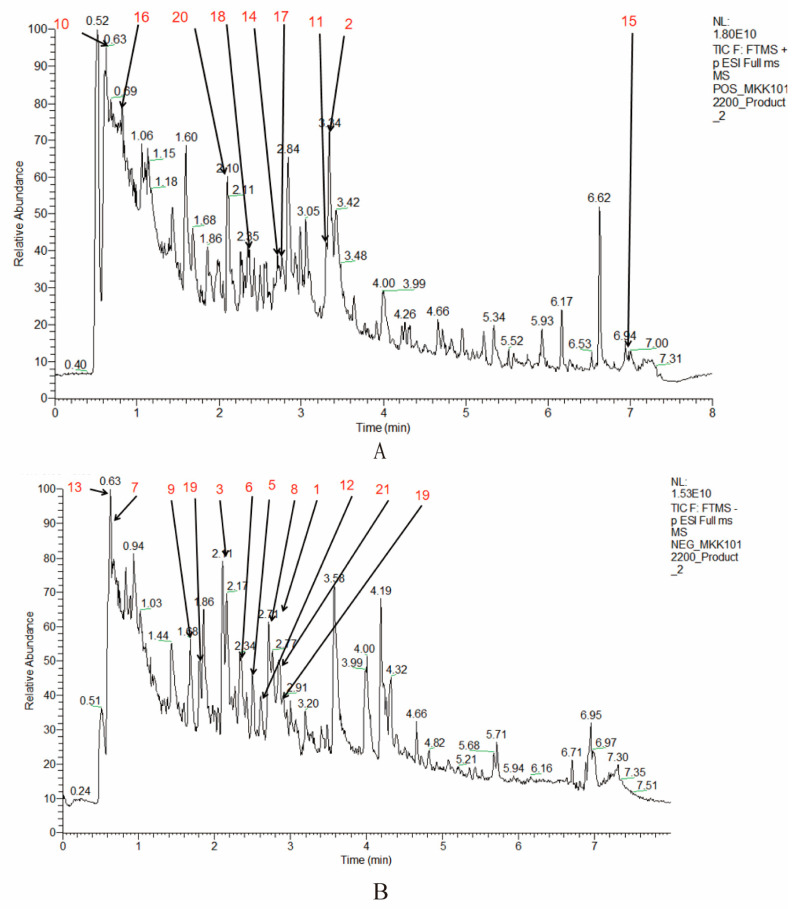
Total ion chromatograms of MELHAC obtained by untargeted LC-MS analysis under two acquisition modes. Multiple characteristic peaks were detected within 0–8 min. (**A**) Positive ino mode (ESI+). (**B**) Negative ino mode(ESI−). The red numbers indicate representative metabolites that were further annotated and summarized in Table 1.

Untargeted LC-MS analysis was performed to characterize the chemical profile of MELHAC. As shown in Figure 1A,B, the total ion chromatograms exhibited multiple well-resolved peaks within the retention time range of 0–8 min, indicating that MELHAC contained structurally diverse metabolites with multiple alkaloids, flavonoids, phenolic acids, and iridoids. In addition, the overall chromatographic patterns obtained under the two acquisition modes (ESI+ and ESI−).

**Table 1:** Representative metabolites identified in MELHAC based on untargeted LC-MS analysis.

	Metabolite	Retention Time	*m*/*z*	Relative Amount
1	Miquelianin	2.7038	477.0676	2.92%
**2**	**Nuciferine**	**3.3472**	**296.1643**	**2.59%**
3	4-caffeoylquinic acid	2.1556	353.0878	2.28%
4	Quercetin-3-o-vicianoside	2.5031	595.1308	1.84%
5	Quercetin 7-glucoside	2.997	463.0886	1.60%
**6**	**chlorogenic acid**	**2.3406**	**353.088**	**1.52%**
7	Galactinol	0.6453	387.1147	1.44%
8	6-Hydroxykaempferol 3-glucoside	2.7191	463.0885	1.39%
9	Kaempferol 3- (2G-apiosylrobinobioside)	1.8478	707.1838	1.01%
10	Trigonelline	0.6241	138.055	0.98%
**11**	**Roemerine**	**3.3172**	**280.1331**	**0.93%**
12	Vitamin p	2.6075	609.1468	0.86%
13	Trehalose	0.6384	341.109	0.83%
14	Morin	2.7178	303.0498	0.80%
15	Oleamide	6.9477	282.279	0.78%
**16**	**1-Deoxynojirimycin**	**0.7453**	**164.0918**	**0.77%**
17	Spiraeoside	2.7178	465.103	0.77%
18	Robinetin	2.5057	303.0498	0.77%
19	Protocatechuic acid	1.7972	153.0184	0.70%
20	1-Caffeoylquinic acid	2.1096	355.1023	0.70%
21	Kaempferol 3-o-galactoside	2.9024	447.0935	0.70%
22	Vanillic acid	2.8358	167.0342	0.70%
23	O-glutarylcarnitine	1.1469	276.1441	0.65%
24	4-Hydroxyisoleucine	0.5971	148.0968	0.54%

Notably, several annotated metabolites, including chlorogenic acid, quercetin derivatives, kaempferol derivatives, and nuciferine, are consistent with the characteristic phytochemical features reported for mulberry leaf, lotus leaf, and Eucommia leaf extracts. Several bolded annotated metabolites, including chlorogenic acid, quercetin derivatives, kaempferol derivatives, and nuciferine, have previously been reported in mulberry leaf, lotus leaf, or Eucommia leaf extracts, suggesting general consistency with the known phytochemical profiles of these plant materials.

### 3.2. Network Pharmacology

**Figure 2:**
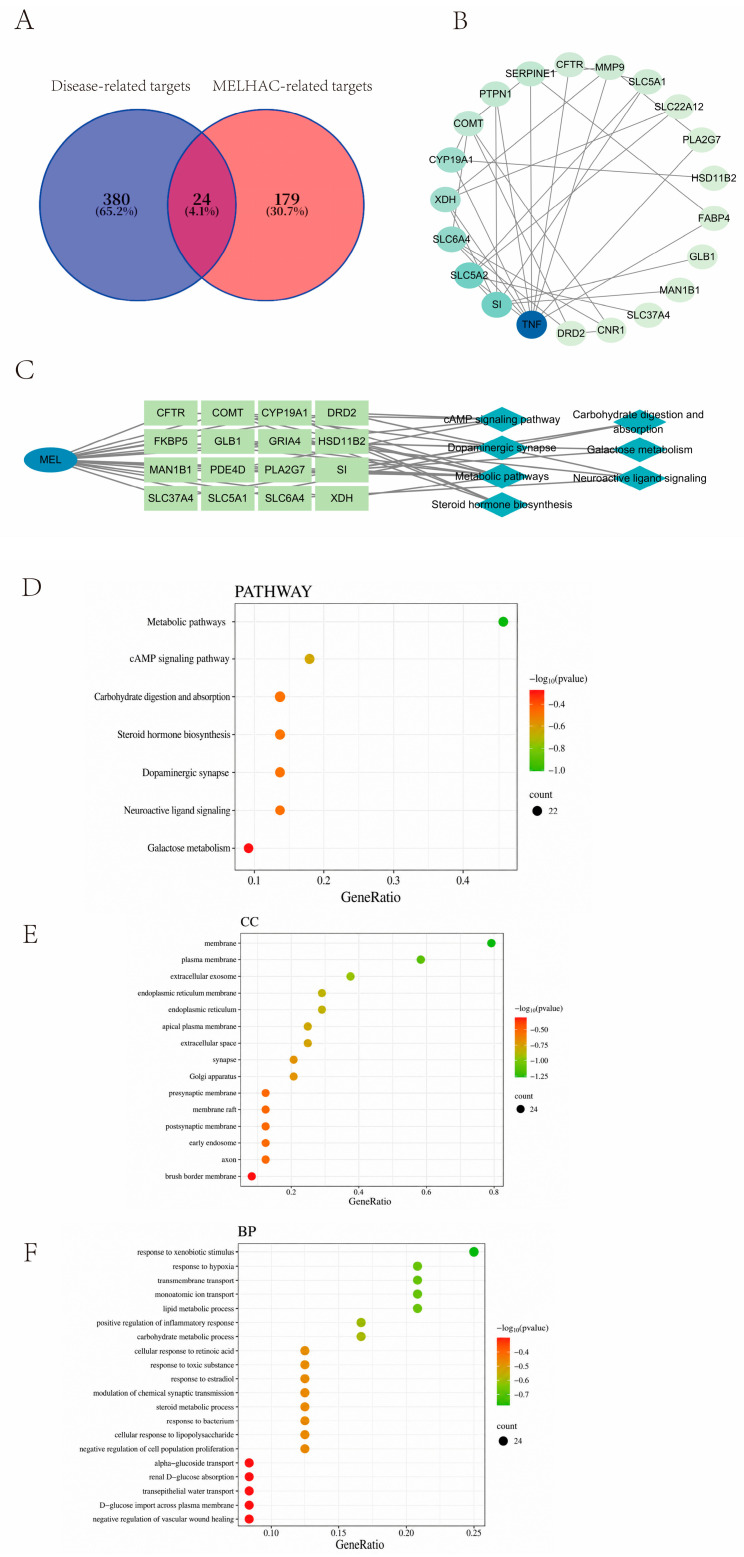
Network pharmacology analysis of MELHAC. (**A**) Venn diagram of abnormal targets and MELHAC targets. (**B**) Interaction network of the overlapping targets. Node color intensity indicates relative network importance based on topological characteristics. (**C**) MELHAC–target–pathway network. (**D**) KEGG pathway enrichment analysis of MELHAC modulated gut microbiota composition and microbial community structure the overlapping targets. (**E**) GO cellular component (CC) enrichment analysis of the overlapping targets. (**F**) GO biological process (BP) enrichment analysis of the overlapping targets. In panels (**D**–**F**), bubble color represents −log10(*p*-value), and bubble size represents the number of enriched targets.

To investigate the potential mechanism by which MELHAC ameliorates glucose and lipid metabolism disorders, the predicted targets of MELHAC were intersected with disease-related abnormal targets. As shown in Figure 2A, 24 overlapping targets were identified from 404 abnormal targets and 203 MELHAC-related targets, suggesting that MELHAC may exert its effects through a multi-target mode of action. A target interaction network was subsequently constructed, revealing relatively close interactions among these shared targets (Figure 2B). Several nodes, including CFTR, COMT, CYP19A1, DRD2, FKBP5, GLB1, GRIA4, HSD11B2, MAN1B1, PDE4D, PLA2G7, SI, SLC37A4, SLC5A1, SLC6A4, and XDH, were identified as potential key targets of MELHAC (Figure 2B,C). KEGG pathway enrichment analysis showed that these targets were mainly enriched in metabolic pathways, the AMPK signaling pathway, carbohydrate digestion and absorption, steroid hormone biosynthesis, dopaminergic synapse, neuroactive ligand–receptor interaction, and galactose metabolism (Figure 2C,D). These findings suggest that MELHAC may regulate energy metabolism, carbohydrate transport and absorption, and endocrine-related signaling pathways to improve metabolic dysfunction. GO enrichment analysis further demonstrated that the overlapping targets were primarily associated with cellular components such as the extracellular region, plasma membrane, extracellular exosome, and endoplasmic reticulum membrane (Figure 2E). In terms of biological processes, these targets were mainly involved in response to xenobiotic stimulus, response to lipid, transmembrane transport, lipid catabolic process, positive regulation of inflammatory response, and carbohydrate metabolic process (Figure 2F). Collectively, these results indicate that MELHAC may improve glucose and lipid metabolism disorders through a coordinated multi-component, multi-target, and multi-pathway regulatory mechanism.

### 3.3. MELHAC Improved Glucose and Lipid Metabolism and Alleviated Histopathological Damage in HFD + Alloxan-Induced Mice

**Figure 3:**
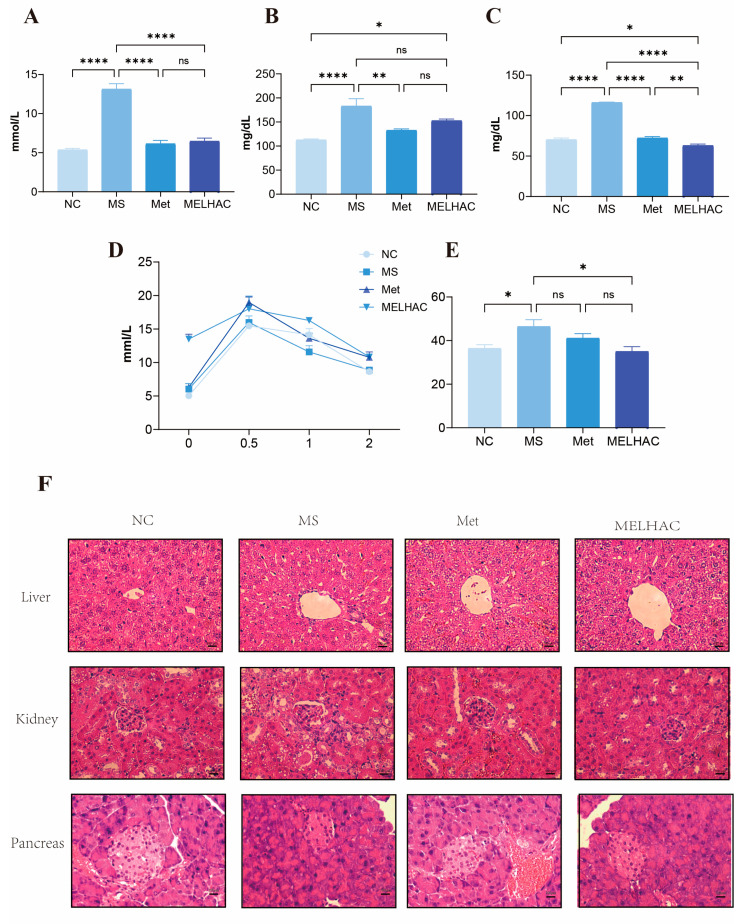
Effects of MELHAC on fasting blood glucose, serum lipid parameters, glucose tolerance, and histopathological changes in HFD + alloxan-induced mice. (**A**) Fasting blood glucose (FBG). (**B**) Serum total cholesterol (TC). (**C**) Serum triglycerides (TG). (**D**) Oral glucose tolerance test (OGTT) curve. (**E**) Area under the curve (AUC) of OGTT. (**F**) Representative hematoxylin and eosin (H&E)-stained sections of liver, kidney, and pancreas from different groups, scale bar = 100 μm. Data are presented as mean ± or SEM. Statistical significance is indicated as ns, not significant; * *p* < 0.05; ** *p* < 0.01; **** *p* < 0.0001.

To evaluate the effects of MELHAC on glucose and lipid metabolism in HFD + alloxan-induced mice, fasting blood glucose, serum lipid parameters, and glucose tolerance were determined. As shown in Figure 3A, fasting blood glucose was markedly elevated in the MS group compared with the NC group, indicating successful establishment of the metabolic disorder model. Treatment with metformin or MELHAC significantly reduced fasting blood glucose relative to the MS group. Similarly, serum TC and TG levels were significantly increased in the MS group, whereas both metformin and MELHAC intervention attenuated these elevations to varying extents (Figure 3B,C), suggesting an improvement in lipid metabolic disturbance.

Glucose tolerance was further assessed by OGTT. As shown in Figure 3D, blood glucose levels in all groups increased rapidly after glucose loading and then gradually declined over time; however, the MS group maintained higher glucose levels throughout the test period than the NC group. In contrast, the Met and MELHAC groups showed lower glucose levels than the MS group, particularly at the later time points, indicating improved glucose clearance. Consistently, the AUC of the OGTT was significantly increased in the MS group, whereas MELHAC administration reduced the AUC compared with the model group (Figure 3E), suggesting an aMELHACioration of glucose intolerance.

To further support the histological interpretation, representative H&E-stained sections were evaluated using a semi-quantitative scoring system, where 0 indicated no obvious lesion, 1 mild lesion, 2 moderate lesion, 3 marked lesion, and 4 severe lesion. Table 2 shows the scores for all slices. The NC group showed scores close to 0 in the liver, kidney, and pancreas. In contrast, the MS group showed higher histopathological scores, with estimated scores of 2 for the liver, 2 for the kidney, and 3 for the pancreas. Compared with the MS group, both Met and MELHAC treatment were associated with lower histopathological scores. The estimated scores in the MELHAC group were approximately 1 for the liver, 1 for the kidney, and 1 for the pancreas, suggesting that MELHAC administration was associated with reduced morphological abnormalities in these tissues.

Collectively, these results suggest that MELHAC improved glucose and lipid metabolic abnormalities and was associated with partial attenuation of liver, kidney, and pancreatic morphological injury in HFD + alloxan-induced mice.

### 3.4. MELHAC Alleviated Hepatic Injury, Oxidative Stress, and Lipid Accumulation in HFD + Alloxan-Induced Mice

**Figure 4:**
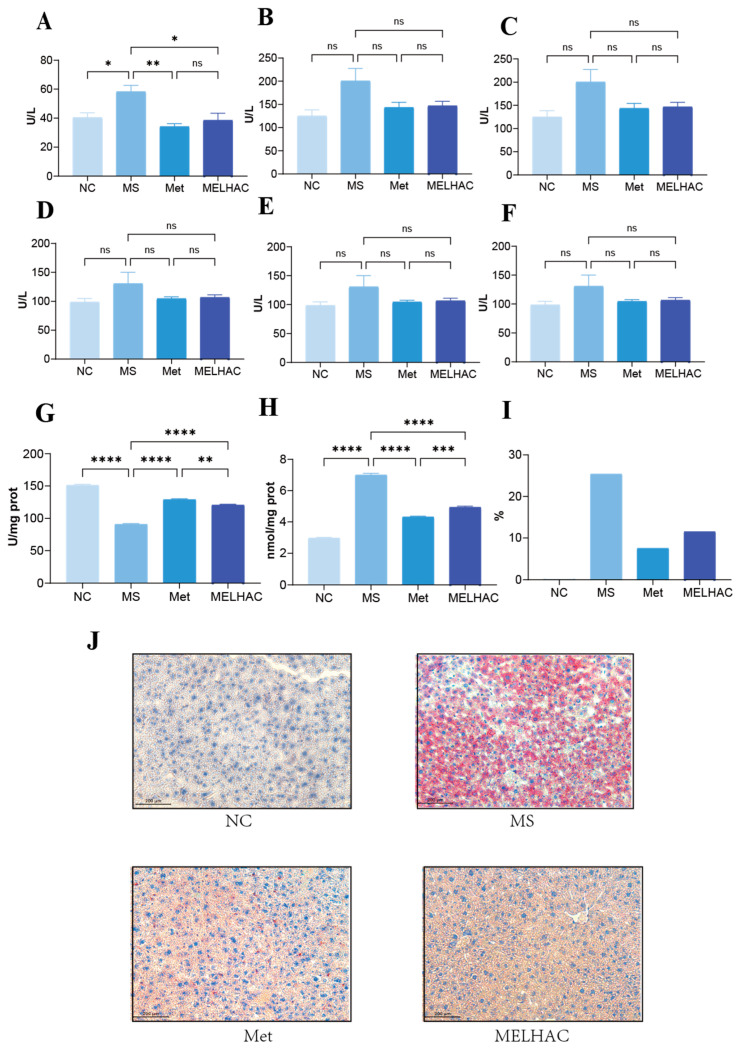
Effects of MELHAC on serum biochemical parameters, hepatic oxidative stress, and lipid accumulation in HFD + alloxan-induced mice. (**A**) Serum alanine aminotransferase (ALT). (**B**) Serum aspartate aminotransferase (AST). (**C**) Serum albumin (ALB). (**D**) Serum alkaline phosphatase (ALP). (**E**) Serum urea (UREA). (**F**) Serum uric acid (UA). (**G**) Hepatic superoxide dismutase (SOD). (**H**) Hepatic malondialdehyde (MDA). (**I**) Quantification of Oil Red O-positive area in liver tissue. (**J**) Representative Oil Red O staining images of liver sections from the NC, MS, Met, and MELHAC groups. Data are presented as mean ± SEM. Statistical significance is indicated as ns, not significant; * *p* < 0.05; ** *p* < 0.01; *** *p* < 0.001; **** *p* < 0.0001. Scale bar = 200 μm.

To evaluate the protective effects of MELHAC on liver injury, oxidative stress, and hepatic lipid deposition in HFD + alloxan-induced mice, serum biochemical parameters, hepatic oxidative stress markers, and Oil Red O staining were analyzed. As shown in Figure 4A,B, serum ALT and AST levels were elevated in the MS group compared with the NC group, indicating liver injury under metabolic stress. Both metformin and MELHAC treatment reduced these elevations to varying degrees. In contrast, no marked changes were observed in ALB, ALP, UREA, or UA among groups, although mild fluctuations were present in the MS group relative to the NC group (Figure 4C–F).

Hepatic oxidative stress was further assessed by measuring SOD and MDA in liver tissue. The MS group showed a pronounced reduction in hepatic SOD compared with the NC group, whereas both metformin and MELHAC treatment restored SOD levels to different extents (Figure 4G). Conversely, hepatic MDA content was markedly increased in the MS group and was decreased after intervention with metformin or MELHAC (Figure 4H), indicating attenuation of hepatic lipid peroxidation.

Consistent with these biochemical findings, Oil Red O staining demonstrated substantial hepatic lipid accumulation in the MS group. Quantitative analysis showed that the Oil Red O-positive area was markedly increased in the model group compared with the NC group, while both the Met and MELHAC groups displayed reduced lipid deposition (Figure 4I). Representative histological images further confirmed minimal lipid staining in the NC group, extensive red-stained lipid droplet accumulation in the MS group, and an obvious reduction in lipid staining in the Met and MELHAC groups (Figure 4J). Collectively, these findings suggest that MELHAC attenuated hepatic oxidative stress and lipid accumulation, accompanied by an improvement in liver injury-related biochemical abnormalities in HFD + alloxan-induced mice.

### 3.5. MELHAC Modulated Gut Microbiota Composition and Microbial Community Structure

**Figure 5:**
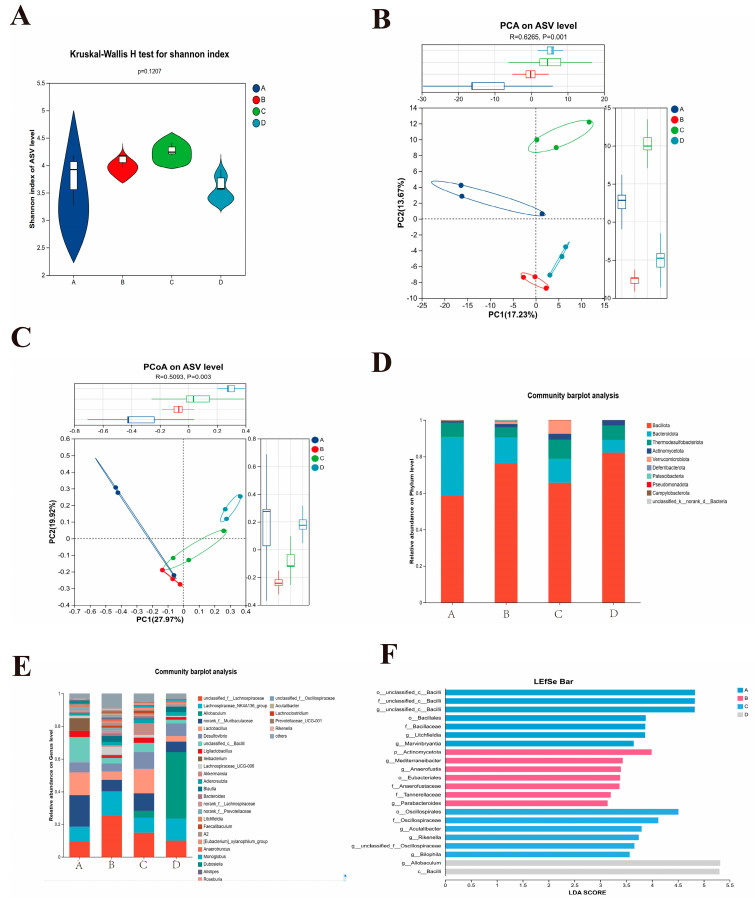
Gut microbial diversity, community structure, taxonomic composition, and differential taxa among the NC (A), MS (B), Met (C), and MELHAC (D) groups. (**A**) Violin plot of the Shannon index at the ASV level. No significant difference in alpha diversity was observed among groups according to the Kruskal–Wallis test. (**B**) PCA plot at the ASV level showing differences in microbial community structure among the four groups. (**C**) PCoA plot at the ASV level showing beta diversity and group-wise separation among the four groups. (**D**) Community bar plot analysis at the phylum level showing the relative abundance of dominant bacterial phyla in each group. (**E**) Community bar plot analysis at the genus level showing the relative abundance of dominant bacterial genera in each group. (**F**) LEfSe bar plot showing the differential microbial taxa enriched in different groups, with the LDA score indicating the effect size of each discriminative feature.

To evaluate the effect of the intervention on gut microbiota, alpha and beta diversity, taxonomic composition, and differential taxa were analyzed among theNC, MS, Met, and MELHAC groups. As shown in Figure 5A, the Shannon index at the ASV level did not differ significantly among the four groups (Kruskal–Wallis test, *p* = 0.1207), suggesting that overall alpha diversity was not markedly altered. In contrast, clear differences in community structure were observed in the beta diversity analyses. PCA showed a distinct separation among groups at the ASV level (R = 0.6265, *p* = 0.001; Figure 5B), and PCoA further confirmed significant group-wise clustering (R = 0.5093, *p* = 0.003; Figure 5C), indicating that microbial community composition was substantially reshaped by modeling and subsequent intervention.

At the phylum level, *Bacillota* and *Bacteroidota* were the dominant phyla in all groups, although their relative abundances varied among groups (Figure 5D). Compared with the NS group, the MS group showed an increased proportion of Bacillota and a reduced proportion of *Bacteroidota*, indicating an altered microbial structure under the disease condition. The MELHAC and Met groups exhibited partial restoration of the phylum-level composition, with shifts in several minor phyla, including *Thermodesulfobacteriota*, *Actinomycetota*, and *Verrucomicrobiota*. At the genus level, the microbial composition also differed markedly among groups (Figure 5E), suggesting that the intervention affected specific bacterial taxa rather than only broad phylum-level distribution.

LEfSe analysis further identified discriminative taxa among groups (Figure 5F). Taxa related to *Bacilli*, *Bacillales*, *Bacillaceae*, *Oscillospirales*, *Oscillospiraceae*, *Acutalibacter*, *Rikenella*, *unclassified_f__Oscillospiraceae*, *Bilophila*, and *Allobaculum* were enriched in one group, whereas *Actinomycetota*, *Mediterraneibacter*, *Anaerofustis*, *Eubacteriales*, *Anaerofustaceae*, *Tannerellaceae*, and *Parabacteroides* were enriched in another group. These findings indicate that the intervention was associated with a marked remodeling of gut microbial structure and the abundance of specific microbial biomarkers, which may contribute to its regulatory effects on host metabolic homeostasis.

### 3.6. MELHAC Showed No Obvious Hematological or Serum Biochemical Toxicity After 10 Days of High-Dose Administration

**Figure 6:**
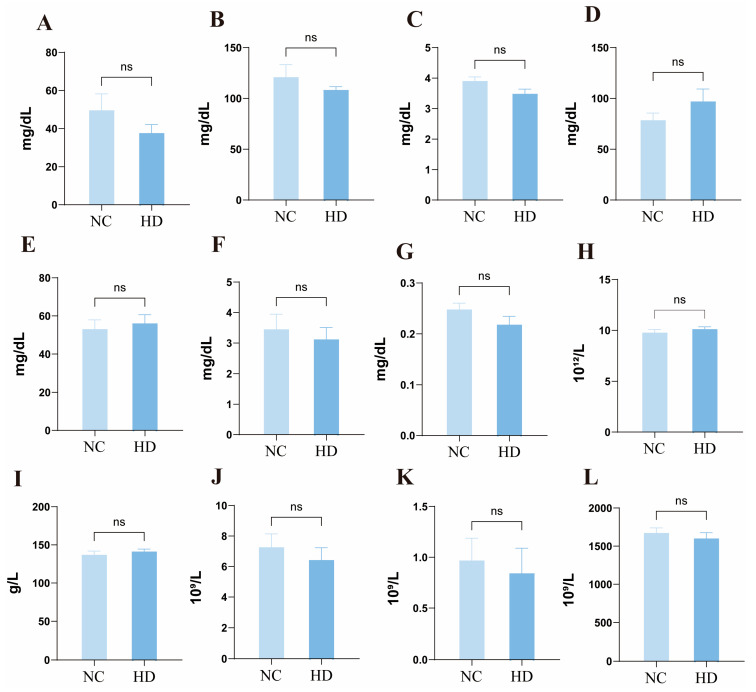
Effects of 10-fold dose administration of MELHAC for 10 days on serum biochemical and hematological parameters in mice. (**A**) Serum alanine aminotransferase (ALT). (**B**) Serum aspartate aminotransferase (AST). (**C**) Serum albumin (ALB). (**D**) Serum alkaline phosphatase (ALP). (**E**) Serum urea (UREA). (**F**) Serum uric acid (UA). (**G**) Creatinine (CREA) (**H**) Red Blood Cell count (RBC) (**I**) Hemoglobin (HGB). (**J**) White blood cell count (WBC). (**K**) Neutrophil count (NEUT). (**L**) Platelet count (PLT). Data are presented as mean ± SEM. Statistical significance is indicated as ns, not significant.

To evaluate the safety of MELHAC, mice were administered a ten-fold equivalent dose for 10 consecutive days. As shown in Figure 6, no significant differences were observed in serum ALT, AST, ALB, ALP, UREA, or UA levels between the normal control (NC) group and the high-dose (HD) group (*p* > 0.05). Similarly, hematological parameters, including hemoglobin (HGB), white blood cell count (WBC), neutrophil count (NEUT), and platelet count (PLT), showed no significant changes following high-dose MELHAC administration (*p* > 0.05). These findings indicate that short-term administration of MELHAC at a ten-fold equivalent dose did not induce detectable hepatic, renal, or hematological toxicity in mice.

### 3.7. MELHAC Showed No Obvious Acute Toxicity in Major Organs After 10 Days

**Figure 7:**
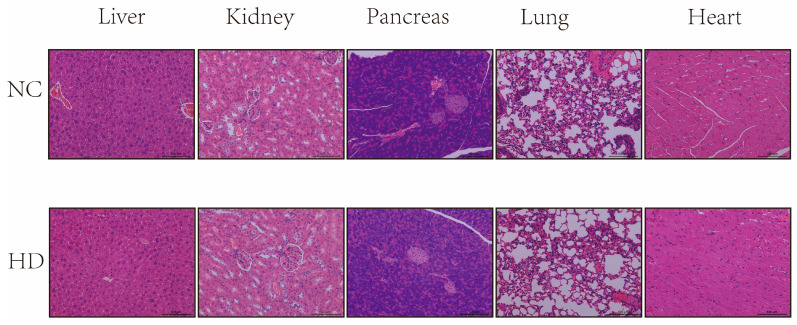
Histopathological evaluation of major organs in the blank control group and the 10× dose group after 10 days of administration, Scale bar = 500 μm.

To preliminarily assess the acute toxicity of MELHAC, histopathological examination of major organs was performed after 10 days of administration at a 10-fold dose. As shown in Figure 7, no obvious treatment-related histopathological abnormalities were observed in the liver, kidney, pancreas, lung, or heart of mice in the 10× dose group compared with the blank control group. The hepatic architecture remained intact, with no apparent hepatocellular degeneration, necrosis, or inflammatory infiltration. Renal tissues showed preserved glomerular and tubular structures without evident pathological injury. The pancreatic islets and surrounding acinar tissues were morphologically comparable between groups. Likewise, no obvious alveolar damage, interstitial thickening, inflammatory infiltration, or myocardial structural abnormalities were observed in the lung or heart tissues. These findings suggest that MELHAC did not induce evident acute toxic effects in major organs under the present experimental conditions.

## 4. Discussion

The present study provides preliminary evidence that MELHAC, a formula composed of mulberry leaf, lotus leaf, and Eucommia leaf extracts, may improve glucose and lipid metabolism disorders in HFD/alloxan-induced mice. MELHAC treatment reduced fasting blood glucose, improved glucose tolerance, decreased serum TC and TG levels, attenuated hepatic lipid accumulation, and improved selected oxidative stress-related parameters. In addition, MELHAC administration was associated with changes in gut microbial community structure and did not induce obvious short-term toxicological abnormalities under the tested high-dose conditions. These findings suggest that MELHAC may have potential as a plant-derived functional food ingredient for metabolic health management.

Untargeted LC–MS analysis indicated that MELHAC contains multiple classes of annotated phytochemicals, including flavonoids, phenolic acids, alkaloids, and iridoids. Several identified metabolites, such as chlorogenic acid, caffeoylquinic acid derivatives, quercetin glycosides, kaempferol glycosides, and nuciferine, are quite similar with the phytochemical features commonly reported in medicinal and edible plant extracts [24,25,26,27,28,29,30,31,32]. These compounds have been associated in previous studies with glucose regulation, lipid metabolism, antioxidant activity, or anti-obesity effects [33,34,35,36]. Therefore, the observed biological effects of MELHAC may be related to the combined contribution of multiple phytochemicals rather than a single active constituent. However, the present LC–MS analysis was mainly qualitative and semi-quantitative. Targeted quantification was not performed, and the exact contribution of individual compounds remains unclear. For future quality control and batch standardization, DNJ, nuciferine, chlorogenic acid, and aucubin may be considered candidate chemical markers, but their quantitative specifications require further validation.

Network pharmacology analysis suggested that MELHAC-related targets may be associated with metabolic pathways, carbohydrate digestion and absorption, steroid hormone biosynthesis, and AMPK-related signaling. AMPK is widely recognized as an important regulator of energy metabolism, glucose utilization, and lipid homeostasis [37,38,39]. Thus, the enrichment of AMPK-related pathways is consistent with the metabolic improvements observed in this study. However, these findings should be interpreted as computational predictions rather than direct mechanistic evidence. AMPK phosphorylation, downstream signaling events, and target-specific molecular responses were not measured in the present study. Therefore, the involvement of AMPK signaling and other predicted pathways remains hypothetical and requires experimental validation.

In the animal model, MELHAC significantly reduced fasting blood glucose and improved glucose tolerance, indicating a beneficial effect on glucose homeostasis. At the same time, MELHAC decreased serum TC and TG levels, suggesting a broader regulatory role in lipid metabolism. Since the HFD + alloxan model combines dietary lipid overload with pancreatic injury, it reflects both insulin-related metabolic imbalance and tissue damage and has been used as an experimental strategy for mimicking features of type 2 diabetes and related metabolic dysfunction [40,41]. The histopathological findings in the liver, kidney, and pancreas supported the biochemical results, showing that MELHAC partially attenuated organ injury under metabolic stress. These data suggest that MELHAC not only affects circulating metabolic markers but also alleviates tissue-level pathological changes.

MELHAC also showed a potential hepatoprotective effect. The MS group exhibited elevated ALT and AST levels, reduced hepatic SOD activity, increased MDA levels, and greater Oil Red O-positive lipid accumulation, whereas MELHAC partially reversed these changes. These findings suggest that MELHAC may improve liver injury-related biochemical abnormalities and hepatic lipid deposition. However, oxidative stress evaluation was limited to SOD and MDA. These markers provide useful but incomplete information regarding antioxidant defense and lipid peroxidation. A broader biomarker panel, including GSH, GSH-Px, CAT, T-AOC, ROS, and inflammatory mediators, should be included in future studies. Similarly, histopathological findings in the current study were mainly qualitative, and blinded pathological scoring would strengthen the objectivity of tissue injury assessment.

Gut microbiota analysis showed that MELHAC treatment was associated with changes in microbial community structure. Although no significant difference was observed in Shannon diversity, PCA and PCoA suggested group-wise separation, and LEfSe analysis identified several discriminative taxa. These findings indicate that MELHAC administration was accompanied by alterations in gut microbial composition. Previous studies have linked gut microbiota to host metabolism through energy harvest, metabolic endotoxemia, bile acid metabolism, short-chain fatty acid production, and inflammatory regulation [16,17,42,43]. However, the current microbiota analysis remains descriptive. Correlation analysis between microbial taxa and metabolic indicators was not performed, and no fecal microbiota transplantation, antibiotic depletion, or germ-free animal experiments were conducted. Therefore, the present data cannot establish whether microbial changes directly contributed to the metabolic benefits of MELHAC or occurred in parallel with them.

The preliminary safety evaluation showed that 10 days of administration at a ten-fold equivalent dose did not cause obvious abnormalities in serum biochemical indices, hematological parameters, or major organ histology. This result supports short-term tolerability under the tested conditions. However, it should not be interpreted as a comprehensive toxicological assessment. Chronic exposure, reproductive toxicity, genotoxicity, bioaccumulation, and long-term organ effects were not evaluated.

Several limitations should be acknowledged. First, only a single MELHAC dose was tested, preventing dose–response evaluation. Second, the sample size was relatively small, with five animals per group, which may limit statistical power and increase the influence of biological variability. Third, insulin-related parameters were not measured. Fourth, predicted molecular mechanisms were not experimentally validated. Fifth, the phytochemical characterization lacked targeted quantitative standardization. Finally, the microbiota and safety findings should be regarded as exploratory and preliminary. Future studies should focus on formulation optimization, active component standardization, dose–response assessment, pharmacokinetic characterization, mechanistic validation, larger animal cohorts, chronic safety evaluation, and ultimately clinical studies. These steps will be essential for advancing MELHAC toward functional food development.

## 5. Conclusions

In conclusion, MELHAC, a formula composed of mulberry leaf, lotus leaf, and eucommia leaf extracts, improved glucose and lipid metabolism in HFD/alloxan-induced mice. MELHAC treatment reduced hyperglycemia and dyslipidemia, alleviated hepatic lipid accumulation, improved oxidative stress-related parameters, and was associated with alterations in gut microbial composition. Network pharmacology analysis further identified several potential targets and pathways that may be involved in the observed biological effects.

However, these findings should be interpreted within the limitations of the present study. Only a single intervention dose was evaluated, insulin-related parameters were not assessed, and the predicted molecular mechanisms were not experimentally validated. In addition, the gut microbiota analysis was primarily descriptive and does not establish a causal relationship between microbial alterations and metabolic improvements. The safety evaluation was limited to short-term exposure and should be regarded as preliminary evidence of tolerability rather than a comprehensive toxicological assessment.

Overall, the present study provides initial evidence supporting the potential of MELHAC as a functional food ingredient for the management of glucose and lipid metabolism disorders. Future studies should focus on dose optimization, active component standardization, pharmacokinetic characterization, mechanistic validation, long-term safety assessment, and clinical evaluation to facilitate its translational development.

## Figures and Tables

**Table 2:** Scores for representative hematoxylin-eosin(H&E) stained sections of the liver, kidneys, and pancreas across different groups.

Tissue	NC	MS	Met	MELHAC
Liver	0	2	1	1
Kidney	0	2	1	1
Pancreas	0	3	1	2

## Data Availability

The original contributions presented in this study are included in the article Further inquiries can be directed to the corresponding authors.

## Abbreviations

The following abbreviations are used in this manuscript:

ALB	albumin
ALP	alkaline phosphatase
ALT	alanine aminotransferase
AMPK	AMP-activated protein kinase
AST	aspartate aminotransferase
ASV	amplicon sequence variant
AUC	area under the curve
BP	biological process
CC	cellular component
CFTR	cystic fibrosis transmembrane conductance regulator
CG	control group
COMT	catechol-O-methyltransferase
CREA	creatinine
CYP19A1	cytochrome P450 family 19 subfamily A member 1
DRD2	dopamine receptor D2
EXP	experimental group
FBG	fasting blood glucose
FKBP5	FK506 binding protein 5
GLB1	galactosidase beta 1
GO	Gene Ontology
GRIA4	glutamate ionotropic receptor AMPA type subunit 4
H&E	hematoxylin and eosin
HDL-C	high-density lipoprotein cholesterol
HFD	high-fat diet
HGB	hemoglobin
HSD11B2	hydroxysteroid 11-beta dehydrogenase 2
KEGG	Kyoto Encyclopedia of Genes and Genomes
LC	liquid chromatography
LC–MS	liquid chromatography–mass spectrometry
LDA	linear discriminant analysis
LDL-C	low-density lipoprotein cholesterol
LEfSe	linear discriminant analysis effect size
MAN1B1	mannosidase alpha class 1B member 1
MDA	malondialdehyde
MELHAC	Mulberry–Eucommia–Lotus Herbal Aqueous Complex
Met	metformin group
MOD	model group
MS	model group
NAFLD	non-alcoholic fatty liver disease
NC	normal control
NEUT	neutrophil count
OGTT	oral glucose tolerance test
PCA	principal component analysis
PCoA	principal coordinates analysis
PDE4D	phosphodiesterase 4D
PLA2G7	phospholipase A2 group VII
PLT	platelet count
POY	positive control group
QC	quality control
RBC	red blood cell count
SI	sucrase-isomaltase
SLC37A4	solute carrier family 37 member 4
SLC5A1	solute carrier family 5 member 1
SLC6A4	solute carrier family 6 member 4
SOD	superoxide dismutase
T2DM	Type 2 Diabetes Mellitus
TC	total cholesterol
TG	triglycerides
UA	uric acid
UHPLC	ultra-high-performance liquid chromatography
UREA	urea
WBC	white blood cell count
XDH	xanthine dehydrogenase
